# A Review of the Immunological Mechanisms Following Mucosal Vaccination of Finfish

**DOI:** 10.3389/fimmu.2015.00427

**Published:** 2015-08-24

**Authors:** Hetron Mweemba Munang’andu, Stephen Mutoloki, Øystein Evensen

**Affiliations:** ^1^Section of Aquatic Medicine and Nutrition, Department of Basic Sciences and Aquatic Medicine, Faculty of Veterinary Medicine and Biosciences, Norwegian University of Life Sciences, Oslo, Norway

**Keywords:** gill, gut, IgM, IgT, mucosal, oral, skin, vaccine

## Abstract

Mucosal organs are principle portals of entry for microbial invasion and as such developing protective vaccines against these pathogens can serve as a first line of defense against infections. In general, all mucosal organs in finfish are covered by a layer of mucus whose main function is not only to prevent pathogen attachment by being continuously secreted and sloughing-off but it serves as a vehicle for antimicrobial compounds, complement, and immunoglobulins that degrade, opsonize, and neutralize invading pathogens on mucosal surfaces. In addition, all mucosal organs in finfish possess antigen-presenting cells (APCs) that activate cells of the adaptive immune system to generate long-lasting protective immune responses. The functional activities of APCs are orchestrated by a vast array of proinflammatory cytokines and chemokines found in all mucosal organs. The adaptive immune system in mucosal organs is made of humoral immune responses that are able to neutralize invading pathogens as well as cellular-mediated immune responses whose kinetics are comparable to those induced by parenteral vaccines. In general, finfish mucosal immune system has the capacity to serve as the first-line defense mechanism against microbial invasion as well as being responsive to vaccination.

## Introduction

Mucosal surfaces are important physical barriers whose main function is to protect the systemic environment of the body against microbial invasion. An ideal mucosal vaccine should have the capacity to produce protective immunity that is able to prevent microbial invasion, colonization, and establishment of infection at portals of entry (1). All mucosal organs are endowed with antigen-presenting cells (APCs) that play a vital role in antigen uptake and processing followed by presentation to naïve B- and T-lymphocytes to induce a long-lasting protective immunity (2). Despite so, our understanding of the immunological basis of mucosal vaccine protection has for a long time lagged behind systemic immunity not only in finfish, but also in higher vertebrates, which has led to a corresponding delay in developing highly protective mucosal vaccines across the vertebrate taxa.

The demand for mucosal vaccines in aquaculture has been exacerbated by different stages of fish production cycles in which administering vaccines by injection might not be feasible thereby rendering the use of mucosal vaccines as an alternative. For Atlantic salmon (*Salmo salar* L), administering vaccines by injection at the freshwater stage is the most commonly applied method while boost vaccination for fish in cages at sea is only applicable by oral vaccination. On the other hand, vaccinating small fish by injection causes stress-related mortalities, which make immersion vaccination a better alternative. While the mode of vaccine delivery is to a large extent dependent on the fish production cycle, developing highly protective vaccines for oral and immersion vaccination has been a serious challenge for a long time because the process of optimizing vaccine delivery methods and measuring immune responses for mucosal vaccines is more complicated than for injectable vaccines (3). For example, the vaccine dose taken up by oral or immersion vaccination is difficult to accurately quantify. Unlike injectable vaccines whose immune response to vaccination is determined by measuring serum antibody levels, there is no optimized quantitative assay established for measuring antibody levels in the mucus of vaccinated fish. Therefore, it is difficult to optimize mucosal vaccine performance for finfish (3). Despite so, gene expression studies show that mucosal vaccines are able to induce immune responses in vaccinated fish. It is anticipated that generating vaccines that have the capacity to induce a combined effect of highly protective mucosal and systemic immune responses could be more effective at attaining sterile immunity. Mucosal immune responses would serve as gatekeepers at the portals of pathogen entry while systemic immunity would serve as a secondary barrier to block the spread of infection to target organs in infected fish.

Considerable progress has been made in optimizating the performance of injectable vaccines in aquaculture (4, 5), but not for mucosal vaccines. However, recent advances in mucosal immunology show that teleosts fish, like all vertebrates, are endowed with a protective immune system although there has been no comprehensive review that puts together a summation of underlying mechanisms of mucosal vaccine protection in finfish, thereby creating the basis for this review. Therefore, this review puts together a collection of different components of the mucosal immune system of finfish with the view to shed insight on how these elements prevent microbial invasion on mucosal surfaces as a basis for designing highly protective mucosal vaccines for finfish.

## Immunological Mechanisms of Vaccine Protection in Different Mucosal Organs

Mucosal organs in finfish have been classified into four broad categories, namely the gut, gills, skin, and nasal mucosa by different scientists (6–9). Hence, in this review, we discuss the immunological basis of mucosal vaccine protection based on this classification.

### Gut mucosal responses to vaccination

The gut immune system of finfish is made of two components, namely the innate and adaptive immune system whose immune responses to vaccination are discussed below.

#### Innate Immune Responses to Mucosal Vaccination in the Gut

A layer of mucus containing antimicrobial peptides, complement factors, immunoglobulins, and other surface defensins covers the mucosal surface of the gut (Table 1). Rombout et al. (6) have recently reviewed the type of immune cells found in the gut of fish. APCs, such as monocytes and macrophages, are found in the lamina propria (LP) while the intra epithelial lymphocytes (IELs) mainly comprises of B and T-lymphocytes. Zhang et al. (10) and Li et al. (11) have shown that teleosts B-cells possess phagocytic properties that play an important role as APCs. It has been shown that IgT coats gut commensal bacteria in a similar pattern as IgA in mammals (10). In addition, Fuglem et al. (12) have reported of M-cells and dendritic-like cells in the gut of salmonid intestinal epithelium.

**Table 1:** **Innate immune components of the gut mucosa in finfish**.

Component	Regulatory/effectors cells/genes	Reference
Mucus and surface defensins	Muc2	(13)
	Antimicrobial peptides	(14, 15)
	Complement system	(16–19)
Cell types	Goblet cells	(14)
	Macrophages	(6, 7)
	Granulocytes	(6, 7)
	Rodlet cells	(20)
	M-cells	(12)
	Enterocytes	(21, 22)
Pattern recognition receptor	Toll-like receptors	(23, 24)
	Peptidoglycan PRR	(25)
Proinflammatory cytokines	IL-1β	(26, 27)
	TNFα	(28)
	IL-6	(27)
Chemokines	CCR6	(29)
	CCR9B	(29)
	CXCL10	(30)
	CK12	(31)

Martin et al. (32) compared the antigen uptake ability of intestinal leukocytes with their head-kidney (HK) and peripheral blood (PBL) counterparts and showed that intestinal phagocytes, when activated, ingested as many yeast cells as their HK counterparts, indicating that gut APCs have the same capacity for antigen uptake as their systemic counterparts. Rombout et al. (21, 22) have shown that there are differences in the distribution of APCs between different gut segments with the second segment having more APCs than the first. Similarly, Fuglem et al. (12) also showed that the uptake of gold-BSA was restricted to dendritic-like cells and other epithelial cells located in mucosal folds found in the second gut segment. In another study, Chen et al. (33) recently showed uptake of inactivated infectious pancreatic necrosis virus (IPNV) antigens in the second gut segment following oral and anal intubation, which were also detected in the HK melanomacrophages of Atlantic salmon (*S. salar* L). Overall, these studies indicate that antigens deposited in the second gut segment were more likely to be taken up by APCs than antigens deposited in other gut segments. Therefore, the challenge is to develop vaccine delivery systems able to deposit antigens in the second gut segment.

Similar to systemic immune responses (5, 34), mucosal antigen uptake is linked to expression of different proinflammatory cytokines, such as IL-1β, TNFα, and IL-8, in the gut (Table 1). In addition, Table 1 shows that chemokines expressed in response to antigen delivery through the gut mucosa are comparable to those induced by parenteral vaccination (35, 36) suggesting that antigen uptake by gut APCs is coordinated by chemokines and cytokines comparable to those induced by systemic antigen delivery systems (35–37). However, there is need for detailed investigations to elucidate the role of these chemokines and cytokines in enhancing the performance of mucosal vaccines in finfish.

#### Adaptive Immune Responses to Mucosal Vaccination in the Gut

All three immunoglobulin (Ig) isotypes characterized in teleosts fish have been detected in the gut (Table 2). Unlike IgM, IgT is specialized in mucosal surfaces in a similar pattern that IgA functions in mammals and it accounts for the largest proportion of the B-cell population found in the gut of finfish (10). Kai et al. (26) have shown that high IgT levels were only expressed by bath or immersion vaccination unlike IgM that had high levels for vaccines administered by injection (4). Vervarcke et al. (38) compared antibody levels induced by anal intubation of vaccines into the second segment of the gut, oral vaccination through feed and intraperitoneal vaccination against *Vibrio anguillarum* and observed high antibody levels in the skin mucus and bile for fish vaccinated by intubation and not in the intraperitoneally vaccinated fish. In addition, they detected high antibody levels for fish vaccinated by intubation than the orally vaccinated fish. They further noted that the antigen dose of vaccines administered by anal intubation correlated with post vaccination antibody levels suggesting that deposition of vaccines in the second segment of the gut by anal intubation produced better correlates of vaccine protection than oral vaccination. In summary, these studies show (i) compartmentalization of mucosal responses in which antigens delivered by intubation into the gut mucosa produced high antibody levels in other mucosal organs, which was not the case for vaccines delivered by the intraperitoneal route, (ii) the second segment of the gut is the most antigen absorptive site able to induce high antibody responses, and (iii) antibodies generated by mucosal vaccination were protective against microbial invasion in the gut mucosa.

**Table 2:** **Adaptive immune components of the gut mucosa in finfish**.

Component	Regulatory/effectors cells/genes	Abbreviation	Reference
Humoral responses	Cell types	B-lymphocytes	(6, 39)
	Immunoglobulins (Ig)	IgT/IgZ	(26, 40–42)
		IgM	(26, 41)
		IgD	(41)
	B-cell transcription factors	Blimp1	(41)
		Pax5	(41)
Cellular responses	Major histocompatibility	MHC-I	(26)
		MHC-II	(26)
	T-helper cells	Th1	(43)
		Th2	(43)
		Treg	(33)
	Transcription	GATA-3	(27, 44–46)
		T-bet	(46–49)
		FoxP3	(33, 45, 49, 50)
	CD4-Regulatory cytokines	IL-2	(27)
		IL-4/13	(27)
		IL-10	(51)
		IL-12	(52, 53)
		IFNγ	(52, 53)
		TNF	(28)
		PD-1	(54)
		TGF-β	(54)
	Cell type	T-lymphocytes	(54, 55)
	CD8 T-cells	CD8α	(26, 32, 54)
	T-cell receptor	TCR genes	(55)
	CD8 transcription factor(s)	Eomesodermin	(47, 48, 56)
	CD8 regulatory cytokines		

Several scientists (32, 57, 58) have shown that CD8α, which is a marker for activated cytotoxic T-lymphocytes (CTLs) in fish, accounts for the largest proportion of T-lymphocytes in the gut of teleosts fish. It is interesting to note that upregulation of CD8α cells has been linked to the corresponding upregulation of MHC-I, suggesting that presentation of antigens via the MHC-I pathway could be linked to activation of naïve CD8 cells into effector CTLs in the gut mucosa of finfish. For example, Kai et al. (26) showed upregulation of MHC-I that corresponded with upregulation of CD8α in the gut of grouper larvae (*Epinephelus coioides*) exposed to nervous necrosis virus (NNV) by oral vaccination, suggesting that activation of CD8α was via the MHC-I pathway. Furthermore, upregulation of T-cell receptor (TCR) and CD3 genes has also been shown to correspond with upregulation of CD8α and MHC-I genes (57, 59, 60). Martin et al. (32) showed a cytotoxic activity that was twice higher in the intestine than in head-kidney leukocytes in rainbow trout while Picchietti et al. (57) showed high cytotoxic activity in lymphocytes purified from the intestinal mucosa, providing further insight into the cellular-mediated activity of activated CTLs in the gut mucosa of finfish. Put together, these studies show that the gut mucosa is endowed with different components of CD8 T-cell responses whose kinetics corresponds with CTL responses shown to eliminate cells infected with intracellular pathogens in higher vertebrates. As for CD4 responses, transcription factors for their specification into Th1 and Th2 subtypes have been characterized in fish and these include T-bet, GATA-3, and FoxP3 (Table 1). Recently, Wang et al. (61) showed that grass carp virus (GCRV) induced upregulation of T-bet when GATA-3 was downregulated in which upregulation of IFNγ correlated with upregulation of T-bet being similar to kinetics induced by parenteral vaccines in finfish (5, 47). Overall, these studies show that the gut mucosa of finfish is endowed with a cellular-mediated immune response orchestrated by CD4 and CD8 genes expressed in response to mucosal vaccination. However, there still remains the challenge of demonstrating the functional role of cellular-mediated immunity in finfish vaccinated by the mucosal route.

### Skin immune responses to vaccination

The skin of teleost fish is endowed with different components of the innate and adaptive immune system, which are responsive to vaccination as shown below.

#### Innate Immune Responses to Mucosal Vaccination in the Skin

Mucus overlying the epidermis is the first line of defense against microbial invasion on the skin surface. It exerts its protective role by being continuously produced and sloughing off to prevent pathogen adherence. Second, it serves as a vehicle for several immune factors that include lysozomes, proteases, alkaline phosphatases, complement, immunoglobulins, lectins, and C-reactive proteins that prevent pathogen invasion into the skin (14, 15, 62–64). Despite so, bath vaccination studies have shown that the primary site of attachment of *V. anguillarum* is the skin in which the bacteria cause local inflammatory responses upon attachment (65, 66). This causes the skin epithelial cells to phagocytose pathogens that attach to the skin surface (7, 65). And as such, different phagocytic cells have been characterized that include macrophages, granulocytes, goblet cells, sacciform cells, club cells, and malpighian cells in finfish (Table 3). In addition, dendritic-like cells have also been characterized from the skin mucosa of different fish species (67–69). The presence of monocytes, macrophages, and dendritic-like cells (67–71) indicates that the skin mucosa is endowed with APCs like other mucosal organs found in finfish. Upregulation of different toll-like receptors (TLRs) after pathogen invasions suggests that cells of the skin mucosa have sensors that are able to detect and bind to pathogen-associated molecular patterns (PAMPs) expressed by invading pathogens (72, 73). Li et al. (74) showed upregulation of TLR2 in orange-spotted grouper (*E. coioides*) exposed to *Cryptocaryon irritans* infection while Zhao et al. (75) showed increased levels of TLR1, 2, and 19 in response to *Ichthyophthirius multifiliis* infection in channel catfish (*Ictalurus punctatus*). It is interesting to note that upregulation of TLRs genes has been shown to correspond with upregulation of proinflammatory genes. For example, Li et al. (74) showed upregulation of TLR2 that coincided with upregulation of IL-1β in orange spotter grouper exposed to *C irritans* pointing to antigen recognition by APCs orchestrated by proinflammatory genes.

**Table 3:** **Innate immune components of the skin mucosa of finfish**.

Component	Regulatory/effectors cells/genes	Reference
Mucus and surface defensins	Muc5B	(13)
	Antimicrobial peptides	(14, 27)
	Complement system	(76)
Cell types	Macrophages	(71, 72)
	Granulocytes	(71, 72)
	Goblet cells	(14, 77, 78)
	Sacciform cells	(78)
	Club cells	(78)
	Malpighian cells	(78, 79)
Pattern recognition receptor	Toll-like receptors	(72, 73)
Proinflammatory cytokines	IL-1β	(27, 80)
	TNFα	(80, 81)
	IL-6	(27, 82)
	IL-8	(27, 80, 81)
Chemokines	CXCR4	(83)
	CXCL10	(30)
	CK1, 3, 9, and 11	(84)

In summary, the skin mucosa of finfish exert its protective mechanisms by (i) having mucus, which prevents pathogen attachment by continuously being produced and sloughing off, (ii) the presence of different host defensins that degrade, opsonize, and neutralize invading pathogens, (iii) carrying out phagocytosis of pathogens that attach onto the skin surface, and (iv) carrying out antigen uptake and processing by APCs that activate the adaptive immune system thereby creating the basis for mucosal vaccination via the skin.

#### Adaptive Immune Responses to Mucosal Vaccination in the Skin

All Ig isotypes characterized in fish have been detected in the skin mucosa (Table 4) and as pointed out by Zhang et al. (10), IgT is the major Ig isotype found in the skin mucosa although IgM is also present but in low quantities. In general, fish antibodies are protective against invading pathogens on mucosal surfaces as shown by Wang et al. (85, 86) and Dickerson and Clark (87) that naive fish exposed to sub-lethal infection of *I. multifiliis* become resistant to subsequent challenge. In their findings, they observed that resistance correlated with antibody levels in sera and skin mucus of immune fish. They further noted that antibodies from resistant fish easily bound to the immobilization antigens (i-antigens) found on the parasite cell and ciliary membranes. Antibody-mediated cross-linking with i-antigens resulted in expulsion of the parasite from exposed fish indicating that mucosal antibodies have the capacity to eliminate pathogens from mucosal surfaces in finfish. Recovered fish were protected from subsequent exposure to the parasite for a long time indicating that sub-lethal infection of *I. multifiliis* could serve as a live vaccine. These studies showed that exposure to *I. multifiliis* infection through the skin mucosa induced both mucosal and systemic antibody responses. In addition, they noted that vaccinating fish using purified i-antigens, provided long-lasting protective immunity against *I. multifiliis*, which makes the i-antigens to be the most promising candidate for subunit vaccine production.

**Table 4:** **Adaptive immune components of the skin mucosa of finfish**.

Component	Regulatory/effectors cells/genes	Abbreviation	Reference
Humoral responses	Cell types	B-lymphocytes	(7, 42, 88)
	Immunoglobulins (Ig)	IgT/IgZ	(7, 40, 42)
		IgM	(27, 76)
		IgD	(7, 8, 10)
Cellular responses	Major histocompatibility	MHC-II	(76, 81)
	T-helper cells	Th17	(89)
		Th2	(90)
		T-bet	(46)
	Transcription	GATA-3	(27, 46, 90)
	CD4-Regulatory cytokines	IL-4/13	(27, 90)
		IL-10	(27)
		IL-12	(91)
		PD-1	(54)
		TGF-β	(81, 82)

In another study, Zhao et al. (92) showed that antibody-secreting cells (ASC), which include B-cells, plasmablasts, and non-replicating plasma cells, were found in low numbers in the skin of channel catfish. However, after immunization against *I. multifiliis* these antibodies increased by 20-fold. Thereafter, the number of ASC in the skin remained high for a long time and they were shown to confer long-term protective immunity against reinfection. However, it is vital to point out that although antibody responses were protective against parasitic infections such as *I. multifiliis*, there are limited studies that show protective immunity against viral and bacterial infections in the skin (93, 94). Hence, there is need for more studies to determine the protective mechanisms of skin antibodies for other pathogens. However, based on studies carried out this far (85–87, 92, 95), indications are that vaccination via the skin mucosa can induce protective antibody responses thereby creating the basis for vaccination by immersion or bath exposure as shown that fish exposed to *I. multifiliis* via the skin were protected by mucosal antibodies against subsequent exposure.

As for cellular-mediated immunity, there are no studies that categorically show the ability of vaccine-induced CD8 T-cell responses to prevent microbial invasion into the skin mucosa of vaccinated fish. In general, only a few studies have been carried out on cellular-mediated immunity found in the skin mucosal of finfish (27, 96). However, it is interesting to note that induction of humoral immune responses due to parasitic flagellate *Ichthyobodo necator* infection correlated with increased CD4^+^ T-cell expression when CD8α was downregulated in the skin of rainbow trout (27). In their study, Chettri et al. (27) observed upregulation of the transcription factor GATA-3 and IL-4/13A, which correlated with upregulation of IgM^+^, suggesting that exposure to *I. necator* induced CD4^+^ responses in the skin mucosa of infected rainbow trout whose kinetics are similar to those induced by inactivated IPNV vaccines in Atlantic salmon (47). In general, these findings demonstrate that the cellular-mediated immune system of the skin mucosa in finfish is responsive to antigen stimulation and that increased CD4^+^ and IL-4/13A levels could play an important role in enhancing antibody functions as shown in mammals (97–99).

### Gill mucosal responses to vaccination

Similar to other mucosal organs in finfish, immune responses to vaccination in the gills are coordinated by different innate and adaptive immune responses as shown below.

#### Innate Immune Responses to Mucosal Vaccination in the Gills of Finfish

Similar to other mucosal organs of finfish, the gill surface is covered by a mucus layer that contains complement, antimicrobial compounds, antibodies, and other surface defensins (Table 5). Different phagocytic cells have been characterized in the gills of different fish species and these include monocytes, macrophages, rodlet cells, eosinophilic granular cells, and neutrophils (Table 5). In addition, dendritic-like cells have also been reported from the gills (100). The expression pattern of CD83, a known surface marker of activated dendritic-like cells in fish (101–103), was shown to correlate with increased uptake of poly(I:C) in the gill mucosa of turbot (*Scophthalmus maximus*) (104), suggesting that dendritic-like cells in finfish express surface markers characteristic of activated APCs after antigen uptake. In addition, upregulation of IL-1β, IL-8, and TNFα has been shown to correspond with upregulation of TLR genes after vaccination in gills of different fish species (23, 25, 74, 105). Furthermore, some studies have also reported of upregulation of chemokines such as CXCL10, CK3, CK9, CK11, and CK12 that correspond with the homing of APCs to antigen deposition sites in the gills (35, 106–108). Put together, these observations show that the gill mucosa in finfish is bestowed with (i) a mucus layer that serves as a vehicle for host defensins, (ii) APCs comprising monocytes, macrophages, and dendritic-like cells, and (iii) regulatory cytokines and chemokines able to coordinate the functional activities of APCs. Therefore, it can be concluded that the gill mucosa is endowed with a functional innate immune system responsive to vaccination able to activate the adaptive immune system.

**Table 5:** **Innate immune components of the gill mucosa of finfish**.

Component	Regulatory/effector cells/genes	Reference
Mucus surface defensins	Muc5B	(13)
	Complement system	(16, 18, 109)
	Antimicrobial peptides	(15, 109, 110)
Cell types	Macrophages	(111, 112)
	Rodlet cells	(20)
	Eosinophilic granulocytes	(111, 113, 114)
	Neutrophils	(111)
Pattern recognition receptor	Toll-like receptors	(23, 72, 92, 111, 114–116)
	Peptidoglycan PR	(25)
Proinflammatory cytokines	IL-1β	(117–121)
	IL-8	(121)
	TNFα	(119–121)

#### Adaptive Immune Responses to Mucosal Vaccination in the Gills of Finfish

Adaptive immune components of the gut identified in finfish are shown in Table 6. Olsen et al. (105) have shown that IgT^+^ B-cells are predominantly located in the epithelial lining of the gill lamellae, suggesting a primary role of this Ig isotype in mucosal defense against pathogen invasion, whereas IgM positive cells are located in gill arterioles and lamellar capillaries. Kai et al. (26) showed that IgM and IgT levels in the gill mucosa were only upregulated by bath or immersion vaccination, suggesting that the route of vaccine delivery had an influence on the induction of humoral immune responses in the gills. Jorgensen et al. (108) showed that IgT and IgM were able to bind to the surface structures of *I. multifiliis* in the gills of immune rainbow trout shortly after invasion. In their studies, they noted that IgT^+^ B-cells were predominantly located in the gill epithelia in the secondary lamellae corresponding to observations made by Olsen et al. (105). Parasites in immune fish were stained with IgT and yet no stain was detected from parasites from non-immune fish. They also observed that IgM^+^ B-cells were only found inside the capillaries of the secondary gill lamellae and yet the parasites located on the gill surface were stained with IgM, suggesting that there is possible diffusion of serum IgM from the systemic compartment to the exterior surface for it to stain the parasites at the mucosal surface within hours after penetration. In summary, these studies show that (i) there is compartmentalization in the distribution of IgT and IgM with the former being on the outer surface of the epithelia lining while the latter being in the capillaries and (ii) both IgM and IgT have the capacity to bind the exterior surfaces of invading pathogens.

**Table 6:** **Adaptive immune components of the gill mucosa of finfish**.

Component	Regulatory/effectors cells/genes	Abbreviation	Reference
Humoral responses	Cell types	B-lymphocytes	(7)
	Immunoglobulins (Ig)	IgT/IgZ	(40, 42, 109, 110)
		IgM	(121–123)
		IgD	(35)
Cellular responses	Major histocompatibility	MHC-I	(119)
		MHC-II	(109, 110, 119)
	T-helper cells	Th1	(46, 124)
		Th2	(90)
		Treg	(45, 49)
	CD4 Transcription factors	GATA-3	(46, 90)
		T-bet	(46, 49, 125)
		FoxP3	(45, 47, 50)
	Chemokines	CCR6	(29)
		CCR7	(35, 107)
		CCR9B	(29)
		CXCL10	(30)
		CK1, 3, 9, 11 and 12	(29)
	CD4-Regulatory cytokines	IL-2	(125)
		IL-4/13	(90)
		IL-10	(124)
		IL-22	(125)
		IFNγ	(125)
		TGF-β	(119)
	Cell type	T-lymphocytes	(124)
	CD8 T-cells	CD8α	(124)
	T-cell receptor	TCR genes	(124)
	CD8 transcription factor(s)	Eomesodermin	(48, 56)
	CD45	CD45	(124)
	CD8 regulatory cytokines		

Several scientists have shown the activation of naïve CD4 and CD8 T-cells in the gill mucosa of different fish species (58, 126, 127). Takazawa et al. (58) showed that CD8α cells account for a large proportion of the total lymphocytes found in the gills of salmonids. It is interesting to note that induction of MHC-I and CD8^+^ responses in the gills is mostly by bath or immersion vaccination (26, 52). Kai et al. (26) and Overgard et al. (52) observed that upregulation of CD8α cells coincided with upregulation of MHC-I after vaccination using viral antigens pointing to possible activation of CD8 cells via the MHC-I pathway. Overgard et al. (53) also showed upregulation of TCR genes alongside upregulation of MHC-I and CD8α pointing to possible binding of TCRs to MHC-I molecules leading to activation of CD8α cells. Moreover, Aquilino et al. (128) showed upregulation of CD3 genes and perforins alongside increased levels of CD8α and MHC-I in rainbow trout exposed to viral hemorrhagic septicemia virus (VHSV). Put together, these studies show that the gill mucosa is endowed with different components of CD8 T-cell mediated immune genes ranging from TCRs that bind to antigens presented on MHC-I molecules to CD3 molecules that activate naïve CD8 T-cells to effector CTLs and perforins that carry out cytotoxicity killing of cells infected with intracellular pathogens. However, there is need for detailed investigations to elucidate the role of CTL responses in mucosal vaccine protection in finfish.

As for CD4 T-cell responses, activation of naïve CD4 T-cells into effector subtypes is via the MHC-II pathway (34). Olsen et al. (105) showed that MHC-II cells were distributed across the gill filaments where they accumulated in the hyperplastic tissue in rainbow trout. As shown in Table 6, different transcription factors that specify the differentiation of naïve CD4 cells into different T-helper (Th) subtypes have been identified and characterized in the gills of different fish species. In addition, different cytokines and chemokines that regulate the functional activities of different Th-cells have also been characterized in different fish species. Studies carried out by Takizawa et al. (90) have shown that the constitutive expression of IL-4/13 and GATA-3 skewed toward a Th2 response in gill cells of isogenic rainbow trout exposed to poly(I:C). In their studies (90), they showed that the kinetics of IL-4/13A and IFNγ in the gill cells were consistent with expression patterns observed from fish vaccinated by injection (47). In general, these studies suggest that CD4 T-cells are responsive to antigen stimulation in the gill mucosa of finfish. Therefore, future studies should seek to identify the helper roles of different Th-subtypes in conferring protective immunity in fish vaccinated via the gill mucosa.

### Nasal mucosal responses to vaccination

Recent advances have shown that the nasal mucosa is endowed with an innate and adaptive immune system comparable to other mucosal organs in finfish (9). Studies carried out by Tacchi et al. (9) have shown that the nasal mucosa expressed different cytokines, chemokines, antimicrobial peptides, complement factors, and TLR genes in rainbow trout vaccinated against *Yersinia ruckeri*. In addition, it also expressed MHC-II genes as well as different B-cell receptor and TCR genes in response to vaccination against *Y. ruckeri*. All three Igs characterized in fish were detected in the nasal mucosa with IgT being the most abundant followed by IgM (9). Tacchi et al. (9) and La Patra et al. (129) showed significant protection against infectious hematopoietic virus infection (IHNV) and *Y ruckeri* in rainbow trout vaccinated via the nasal mucosa after challenge. In general, indications are that the nasal mucosa uses similar protective mechanisms with those used by other mucosal organs in finfish. However, given that the nasal mucosa is a recently discovered mucosal organ whose protective mechanisms have only been reported in rainbow trout, there is need for follow-up studies in order to consolidate these findings in other fish species.

## General Discussion and Conclusion

In general, teleosts fish are endowed with different mucosal organs that include the gills, gut, skin, and nasal mucosa (6, 7, 9, 10). It is interesting to note that all mucosal organs have an innate immune systems, which is made of three important elements, namely (i) mucus whose function is not only to prevent microbial attachment to mucosal surfaces by continuously being excreted and sloughed-off but it serves as a vehicle for antimicrobial peptides, complement, and Igs that degrade, opsonize, and neutralize invading pathogens on mucosal surfaces; (ii) APCs that carry out antigen uptake, processing, and presentation to cells of the adaptive immune systems (130); and finally (iii) proinflammatory cytokines and chemokines that coordinate the functional activities of APCs. Overall, this review shows that the innate immune systems in different mucosal organs is responsive to mucosal vaccination and that it has the capacity to activate cells of the adaptive immune systems in finfish.

It is interesting to note that all three Ig isotypes characterized in finfish have been detected in all mucosal organs (6, 7, 9, 10). Based on studies carried out this far, it is evident that mucosal antibody responses are the only component of the adaptive immune system that has been shown to confer protective immunity in vaccinated fish (85, 86, 131) although the majority of studies that support this observation are based on the neutralizing ability of mucosal antibodies on parasitic infections (85, 86, 131) with only a few studies showing protection against viral and bacterial infections (93, 94). Nevertheless, it is interesting to note that there is compartmentalization in the expression of different Ig isotypes with IgT being the major isotype in mucosal organs while IgM is mostly found in systemic circulation (7, 10, 40). In addition, there is compartmentalization in terms of the physical distribution of IgT and IgM in some mucosal organs as shown that in the gills IgT is mainly found on exterior surfaces of the gill lamellae (105, 131), suggesting that this isotypes functions as a gatekeeper at the portals of entry whereas IgM is mostly found in the arterioles (105, 131), suggesting that its role is to prevent systemic dissemination of pathogens as a secondary defense strategy in situation where IgT on mucosal surfaces fails to prevent the penetration of pathogens into the systemic environment.

Although cellular-mediated immunity is present in all mucosal organs, its existence is mostly demonstrated by gene expression. The ability of these genes to prevent microbial invasion in vaccinated fish has not been clearly elucidated although the kinetics of CD4^+^ and CD8^+^ gene expression in different mucosal organs suggests that cellular-mediated immunity is responsive to vaccination in finfish. And as pointed out in our previous studies (5, 34, 35), the route of antigen delivery into APCs is deterministic of the type of cellular-mediated immune response induced by vaccination. Antigens delivered via the intracellular route evoke both CD4^+^ and CD8^+^ responses whereas antigens delivered by the extracellular route only induce CD4^+^ responses. And as pointed out by Howarth and Elliot (132), the most protective vaccines are those that stimulate both CD4^+^ and CD8^+^ responses. Therefore, live virus and DNA vaccines that evoke both CD4^+^ and CD8^+^ responses are likely to produce better protection than inactivated vaccines. Although live vaccines produce long-lasting protective immunity given that they are replicative and are more immunogenic than in inactivated vaccines (133, 134), they are less used in aquaculture because of the fear of reversion to virulence as shown in the case of IPNV that avirulent strains can revert to virulence under stress conditions (135). On the other hand, DNA vaccines that are also delivered via the intracellular route are not widely used in aquaculture for ethical reasons because of their genetic modified nature (34).This far, only the IHNV-DNA vaccine has been licensed for commercial use in Canada. Hence, the majority of commercial vaccines currently used in aquaculture are inactivated vaccines, which are limited to induction of CD4^+^ and humoral immune responses. Hence, there still remains the challenge of developing intracellular antigens delivery systems able to evoke of CD4^+^ and CD8^+^ T-cell responses that do not pose the danger of reversion to virulence.

This review summarizes the different components of the innate and adaptive immune systems in mucosal tissues of finish, which are activated following mucosal infection and/or vaccination studies. Based on the synopsis put forth in this review, mucosal antibodies appear to play a key role in conferring protective immunity in vaccinated fish (85, 86, 92, 93, 95). On the other hand, there still remains the challenge of elucidating the ability of mucosal vaccine-induced cellular-mediated immune responses to protect fish against microbial invasion in mucosal organs.

## Conflict of Interest Statement

The authors declare that the research was conducted in the absence of any commercial or financial relationships that could be construed as a potential conflict of interest.

## References

[1] Medina E Guzman CA. Modulation of immune responses following antigen administration by mucosal route. FEMS Immunol Med Microbiol (2000) 27(4):305–11. 10.1111/j.1574-695X.2000.tb01444.x 10727886

[2] Ogra PL Faden H Welliver RC. Vaccination strategies for mucosal immune responses. Clin Microbiol Rev (2001) 14(2):430. 10.1128/CMR.14.2.430-445.2001 11292646 PMC88982

[3] Neutra MR Kozlowski PA. Mucosal vaccines: the promise and the challenge. Nat Rev Immunol (2006) 6(2):148–58. 10.1038/nri1777 16491139

[4] Munang’andu HM Fredriksen BN Mutoloki S Dalmo RA Evensen O. Antigen dose and humoral immune response correspond with protection for inactivated infectious pancreatic necrosis virus vaccines in Atlantic salmon (*Salmo salar* L). Vet Res (2013) 44:7. 10.1186/1297-9716-44-7 23398909 PMC3668999

[5] Munang’andu HM Mutoloki S Evensen O. Acquired immunity and vaccination against infectious pancreatic necrosis virus of salmon. Dev Comp Immunol (2014) 43(2):184–96. 10.1016/j.dci.2013.08.008 23962742

[6] Rombout JHWM Abelli L Picchietti S Scapigliati G Kiron V. Teleost intestinal immunology. Fish Shellfish Immunol (2011) 31(5):616–26. 10.1016/j.fsi.2010.09.001 20832474

[7] Salinas I Zhang YA Sunyer JO. Mucosal immunoglobulins and B cells of teleost fish. Dev Comp Immunol (2011) 35(12):1346–65. 10.1016/j.dci.2011.11.009 22133710 PMC3428141

[8] Xu Z Parra D Gómez D Salinas I Zhang YA von Gersdorff Jørgensen L Teleost skin, an ancient mucosal surface that elicits gut-like immune responses. Proc Natl Acad Sci U S A (2013) 110(32):13097–102. 10.1073/pnas.1304319110 23884653 PMC3740891

[9] Tacchi L Musharrafieh R Larragoite ET Crossey K Erhardt EB Martin SA Nasal immunity is an ancient arm of the mucosal immune system of vertebrates. Nat Commun (2014) 5:5205. 10.1038/ncomms6205 25335508 PMC4321879

[10] Zhang YA Salinas I Li J Parra D Bjork S Xu Z IgT, a primitive immunoglobulin class specialized in mucosal immunity. Nat Immunol (2010) 11(9):827–U82. 10.1038/ni.1913 20676094 PMC3459821

[11] Li J Barreda DR Zhang YA Boshra H Gelman AE Lapatra S B lymphocytes from early vertebrates have potent phagocytic and microbicidal abilities. Nat Immunol (2006) 7(10):1116–24. 10.1038/ni1389 16980980

[12] Fuglem B Jirillo E Bjerkås I Kiyono H Nochi T Yuki Y Antigen-sampling cells in the salmonid intestinal epithelium. Dev Comp Immunol (2010) 34(7):768–74. 10.1016/j.dci.2010.02.007 20178814

[13] Marel MV Adamek M Gonzalez SF Frost P Rombout JH Wiegertjes GF Molecular cloning and expression of two beta-defensin and two mucin genes in common carp (*Cyprinus carpio* L.) and their up-regulation after beta-glucan feeding. Fish Shellfish Immunol (2012) 32(3):494–501. 10.1016/j.fsi.2011.12.008 22227003

[14] Cole AM Darouiche RO Legarda D Connell N Diamond G. Characterization of a fish antimicrobial peptide: gene expression, subcellular localization, and spectrum of activity. Antimicrob Agents Chemother (2000) 44(8):2039–45. 10.1128/AAC.44.8.2039-2045.2000 10898673 PMC90011

[15] Gomez D Sunyer JO Salinas I. The mucosal immune system of fish: the evolution of tolerating commensals while fighting pathogens. Fish Shellfish Immunol (2013) 35(6):1729–39. 10.1016/j.fsi.2013.09.032 24099804 PMC3963484

[16] Løvoll M Fischer U Mathisen GS Bøgwald J Ototake M Dalmo RA. The C3 subtypes are differentially regulated after immunostimulation in rainbow trout, but head kidney macrophages do not contribute to C3 transcription. Vet Immunol Immunopathol (2007) 117(3–4):284–95. 10.1016/j.vetimm.2007.03.005 17449114

[17] Løvoll M Johnsen H Boshra H Bøgwald J Sunyer JO Dalmo RA. The ontogeny and extrahepatic expression of complement factor C3 in Atlantic salmon (*Salmo salar*). Fish Shellfish Immunol (2007) 23(3):542–52. 10.1016/j.fsi.2007.01.002 17449276

[18] Lange SR Bambir S Dodds AW Magnadottir B. The ontogeny of complement component C3 in Atlantic cod (*Gadus morhua* L.) – an immunohistochemical study. Fish Shellfish Immunol (2004) 16(3):359–67. 10.1016/j.fsi.2003.06.001 15123303

[19] Lange S Bambir S Dodds AW Magnadottir B. An immunohistochemical study on complement component C3 in juvenile Atlantic halibut (*Hippoglossus hippoglossus* L.). Dev Comp Immunol (2004) 28(6):593–601. 10.1016/j.dci.2003.10.006 15177113

[20] Bielek E. Rodlet cells in teleosts: new ultrastructural observations on the distribution of the cores in trout (*Oncorhynchus mykiss*, *Salmo trutta* L.). J Submicrosc Cytol Pathol (2002) 34(3):271–8. 12408360

[21] Rombout JHWM Lamers CHJ Helfrich MH Dekker A Tavernethiele JJ. Uptake and transport of intact macromolecules in the intestinal epithelium of carp (*Cyprinus carpio* L) and the possible immunological implications. Cell Tissue Res (1985) 239(3):519–30. 10.1007/BF00219230 3986879

[22] Rombout JHWM Vandenberg AA Uptake and transport of ferritin in the epithelium of carp (*Cyprinus carpio* L) and the possible immunological implications. Cell Biol Int Rep (1985) 9(6):516 10.1016/0309-1651(85)90003-7 4028172

[23] Su JG Yang CR Xiong F Wang YP Zhu ZY. Toll-like receptor 4 signaling pathway can be triggered by grass carp reovirus and *Aeromonas hydrophila* infection in rare minnow *Gobiocypris rarus*. Fish Shellfish Immunol (2009) 27(1):33–9. 10.1016/j.fsi.2009.02.016 19264133

[24] Takano T Kondo H Hirono I Endo M Saito-Taki T Aoki T. Molecular cloning and characterization of toll-like receptor 9 in Japanese flounder, *Paralichthys olivaceus*. Mol Immunol (2007) 44(8):1845–53. 10.1016/j.molimm.2006.10.018 17118454

[25] Sun L Liu S Wang R Li C Zhang J Liu Z. Pathogen recognition receptors in channel catfish: IV. Identification, phylogeny and expression analysis of peptidoglycan recognition proteins. Dev Comp Immunol (2014) 46(2):291–9. 10.1016/j.dci.2014.04.018 24814805

[26] Kai YH Wu YC Chi SC. Immune gene expressions in grouper larvae (*Epinephelus coioides*) induced by bath and oral vaccinations with inactivated betanodavirus. Fish Shellfish Immunol (2014) 40(2):563–9. 10.1016/j.fsi.2014.08.005 25130145

[27] Chettri JK Kuhn JA Jaafar RM Kania PW Møller OS Buchmann K. Epidermal response of rainbow trout to *Ichthyobodo necator*: immunohistochemical and gene expression studies indicate a Th1-/Th2-like switch. J Fish Dis (2014) 37(9):771–83. 10.1111/jfd.12169 23952070

[28] Buchmann K. Immune mechanisms in fish skin against monogeneans – a model. Folia Parasitol (1999) 46(1):1–9. 10408955

[29] Dixon B Luque A Abós B Castro R González-Torres L Tafalla C. Molecular characterization of three novel chemokine receptors in rainbow trout (*Oncorhynchus mykiss*). Fish Shellfish Immunol (2013) 34(2):641–51. 10.1016/j.fsi.2012.12.003 23257202

[30] Baoprasertkul P Peatman E Chen L He C Kucuktas H Li P Sequence analysis and expression of a CXC chemokine in resistant and susceptible catfish after infection of *Edwardsiella ictaluri*. Dev Comp Immunol (2004) 28(7–8):769–80. 10.1016/j.dci.2003.12.002 15043945

[31] Montero J Ordas MC Alejo A Gonzalez-Torres L Sevilla N Tafalla C. CK12, a rainbow trout chemokine with lymphocyte chemo-attractant capacity associated to mucosal tissues. Mol Immunol (2011) 48(9–10):1102–13. 10.1016/j.molimm.2011.02.005 21388685

[32] Martin E Trichet VV Legrand-Frossi C Frippiat JP. Comparison between intestinal and non-mucosal immune functions of rainbow trout, *Oncorhynchus mykiss*. Fish Shellfish Immunol (2012) 33(6):1258–68. 10.1016/j.fsi.2012.09.019 23026718

[33] Chen L Klaric G Wadsworth S Jayasinghe S Kuo TY Evensen Ø Augmentation of the antibody response of Atlantic salmon by oral administration of alginate-encapsulated IPNV antigens. PLoS One (2014) 9(10):e109337. 10.1371/journal.pone.0109337 25310804 PMC4195674

[34] Munang’andu HM Evensen O. A review of intra- and extracellular antigen delivery systems for virus vaccines of finfish. J Immunol Res (2015) 2015:960859. 10.1155/2015/960859 26065009 PMC4433699

[35] Castro R Bromage E Abós B Pignatelli J González Granja A Luque A CCR7 is mainly expressed in teleost gills, where it defines an IgD(+)IgM(-) B lymphocyte subset. J Immunol (2014) 192(3):1257–66. 10.4049/jimmunol.1302471 24353268

[36] Jimenez N Coll J Salguero FJ Tafalla C. Co-injection of interleukin 8 with the glycoprotein gene from viral haemorrhagic septicemia virus (VHSV) modulates the cytokine response in rainbow trout (*Oncorhynchus mykiss*). Vaccine (2006) 24(27–28):5615–26. 10.1016/j.vaccine.2006.04.061 16725233

[37] Holvold LB Fredriksen BN Bogwald J Dalmo RA. Transgene and immune gene expression following intramuscular injection of Atlantic salmon (*Salmo salar* L.) with DNA-releasing PLGA nano- and microparticles. Fish Shellfish Immunol (2013) 35(3):890–9. 10.1016/j.fsi.2013.06.030 23850547

[38] Vervarcke S Ollevier F Kinget R Michoel A. Mucosal response in African catfish after administration of *Vibrio anguillarum* O2 antigens via different routes. Fish Shellfish Immunol (2005) 18(2):125–33. 10.1016/j.fsi.2004.06.004 15475309

[39] Zhang YA Salinas I Sunyer JO. Recent findings on the structure and function of teleost IgT. Fish Shellfish Immunol (2011) 31(5):627–34. 10.1016/j.fsi.2011.03.021 21466854 PMC3404837

[40] Hansen JD Landis ED Phillips RB. Discovery of a unique Ig heavy-chain isotype (IgT) in rainbow trout: implications for a distinctive B cell developmental pathway in teleost fish. Proc Natl Acad Sci U S A (2005) 102(19):6919–24. 10.1073/pnas.0500027102 15863615 PMC1100771

[41] Ballesteros NA Castro R Abos B Rodríguez Saint-Jean SS Pérez-Prieto SI Tafalla C. The pyloric caeca area is a major site for IgM(+) and IgT(+) B Cell recruitment in response to oral vaccination in rainbow trout. PLoS One (2013) 8(6):e66118. 10.1371/journal.pone.0066118 23785475 PMC3681912

[42] Hu YL Xiang LX Shao JZ. Identification and characterization of a novel immunoglobulin Z isotype in zebrafish: implications for a distinct B cell receptor in lower vertebrates. Mol Immunol (2010) 47(4):738–46. 10.1016/j.molimm.2009.10.010 19931913

[43] Kumari J Bogwald J Dalmo RA Vaccination of Atlantic salmon, *Salmo salar* L., with *Aeromonas salmonicida* and infectious pancreatic necrosis virus (IPNV) showed a mixed Th1/Th2/Treg response. J Fish Dis (2013) 36(10):881–6. 10.1111/jfd.12100 23521564

[44] Kumari J Bogwald J Dalmo RA. Transcription factor GATA-3 in Atlantic salmon (*Salmo salar*): molecular characterization, promoter activity and expression analysis. Mol Immunol (2009) 46(15):3099–107. 10.1016/j.molimm.2009.06.008 19576635

[45] Zhang ZB Chi H Niu CJ Bogwald J Dalmo RA. Molecular cloning and characterization of Foxp3 in Atlantic salmon (*Salmo salar*). Fish Shellfish Immunol (2011) 30(3):902–9. 10.1016/j.fsi.2011.01.012 21276855

[46] Wang TH Holland JW Martin SAM Secombes CJ. Sequence and expression analysis of two T helper master transcription factors, T-bet and GATA3, in rainbow trout *Oncorhynchus mykiss* and analysis of their expression during bacterial and parasitic infection. Fish Shellfish Immunol (2010) 29(5):705–15. 10.1016/j.fsi.2010.06.016 20633655

[47] Munang’andu HM Fredriksen BN Mutoloki S Dalmo RA Evensen O. The kinetics of CD4+and CD8+T-cell gene expression correlate with protection in Atlantic salmon (*Salmo salar* L) vaccinated against infectious pancreatic necrosis. Vaccine (2013) 31(15):1956–63. 10.1016/j.vaccine.2013.02.008 23422142

[48] Takizawa F Araki K Ohtani M Toda H Saito Y Lampe VS Transcription analysis of two Eomesodermin genes in lymphocyte subsets of two teleost species. Fish Shellfish Immunol (2014) 36(1):215–22. 10.1016/j.fsi.2013.11.004 24239596

[49] Mitra S Alnabulsi A Secombes CJ Bird S. Identification and characterization of the transcription factors involved in T-cell development, t-bet, stat6 and foxp3, within the zebrafish, *Danio rerio*. FEBS J (2010) 277(1):128–47. 10.1111/j.1742-4658.2009.07460.x 19961539

[50] Wang T Monte MM Huang W Boudinot P Martin SA Secombes CJ. Identification of two FoxP3 genes in rainbow trout (*Oncorhynchus mykiss*) with differential induction patterns. Mol Immunol (2010) 47(16):2563–74. 10.1016/j.molimm.2010.06.015 20684994

[51] Skugor S Glover KA Nilsen F Krasnov A. Local and systemic gene expression responses of Atlantic salmon (*Salmo salar* L.) to infection with the salmon louse (*Lepeophtheirus salmonis*). BMC Genomics (2008) 9:498. 10.1186/1471-2164-9-498 18945374 PMC2582245

[52] Overgard AC Patel S Nostbakken OJ Nerland AH. Atlantic halibut (*Hippoglossus hippoglossus* L.) T-cell and cytokine response after vaccination and challenge with nodavirus. Vaccine (2013) 31(19):2395–402. 10.1016/j.vaccine.2013.01.034 23370152

[53] Overgard AC Nerland AH Fiksdal IU Patel S. Atlantic halibut experimentally infected with nodavirus shows increased levels of T-cell marker and IFNgamma transcripts. Dev Comp Immunol (2012) 37(1):139–50. 10.1016/j.dci.2011.10.003 22020051

[54] Buonocore F Castro R Randelli E Lefranc MP Six A Kuhl H Diversity, molecular characterization and expression of T cell receptor gamma in a teleost tish, the sea bass (*Dicentrarchus labrax*, L). PLoS One (2012) 7(10): e47957. 10.1371/journal.pone.0047957 23133531 PMC3485050

[55] Marozzi C Randelli E Buonocore F Scapigliati G T cells from spleen, intestine, and gills of sea bass *Dicentrarchus labrax*. Fish Shellfish Immunol (2013) 34(6):1664 10.1016/j.fsi.2013.03.094

[56] Kumari J Bogwald J Dalmo RA. Eomesodermin of Atlantic salmon: an important regulator of cytolytic gene and interferon gamma expression in spleen lymphocytes. PLoS One (2013) 8(2):e55893. 10.1371/journal.pone.0055893 23409078 PMC3567031

[57] Picchietti S Guerra L Bertoni F Randelli E Belardinelli MC Buonocore F Intestinal T cells of *Dicentrarchus labrax* (L.): gene expression and functional studies. Fish Shellfish Immunol (2011) 30(2):609–17. 10.1016/j.fsi.2010.12.006 21168509

[58] Takizawa F Dijkstra JM Kotterba P Korytář T Kock H Köllner B The expression of CD8 alpha discriminates distinct T cell subsets in teleost fish. Dev Comp Immunol (2011) 35(7):752. 10.1016/j.dci.2011.02.008 21352850

[59] Boschi I Randelli E Buonocore F Casani D Bernini C Fausto AM Transcription of T cell-related genes in teleost fish, and the European sea bass (*Dicentrarchus labrax*) as a model. Fish Shellfish Immunol (2011) 31(5):655–62. 10.1016/j.fsi.2010.10.001 20950688

[60] Randelli E Scala V Casani D Costantini S Facchiano A Mazzini M T cell receptor beta chain from sea bream (*Sparus aurata*): molecular cloning, expression and modelling of the complexes with MHC class I. Mol Immunol (2008) 45(7):2017–27. 10.1016/j.molimm.2007.10.027 18035419

[61] Wang L Shang N Feng H Guo QL Dai HP. Molecular cloning of grass carp (*Ctenopharyngodon idellus*) T-bet and GATA-3, and their expression profiles with IFN-gamma in response to grass carp reovirus (GCRV) infection. Fish Physiol Biochem (2013) 39(4):793–805. 10.1007/s10695-012-9741-y 23108805

[62] Guardiola FA Cuesta A Meseguer J Esteban MA Constitutive humoral innate defence mechanisms present in epidermal mucus of gilthead seabream (*Sparus aurata* L.). Fish Shellfish Immunol (2013) 34(6):1709 10.1016/j.fsi.2013.03.222

[63] Guardiola FA Cuesta A Arizcun M Meseguer J Esteban MA. Comparative skin mucus and serum humoral defence mechanisms in the teleost gilthead seabream (*Sparus aurata*). Fish Shellfish Immunol (2014) 36(2):545–51. 10.1016/j.fsi.2014.01.001 24412437

[64] Woo PT. Protective immunity in fish against protozoan diseases. Parassitologia (2007) 49(3):185–91. 18410078

[65] Lindell K Fahlgren A Hjerde E Willassen NP Fällman M Milton DL. Lipopolysaccharide O-antigen prevents phagocytosis of *Vibrio anguillarum* by rainbow trout (*Oncorhynchus mykiss*) skin epithelial cells. PLoS One (2012) 7(5):e37678. 10.1371/journal.pone.0037678 22662189 PMC3360773

[66] Rajan B Lokesh J Kiron V Brinchmann MF. Differentially expressed proteins in the skin mucus of Atlantic cod (*Gadus morhua*) upon natural infection with *Vibrio anguillarum*. BMC Vet Res (2013) 9:103. 10.1186/1746-6148-9-103 23672475 PMC3666997

[67] Bassity E Clark TG. Functional identification of dendritic cells in the teleost model, rainbow trout (*Oncorhynchus mykiss*). PLoS One (2012) 7(3):e33196. 10.1371/journal.pone.0033196 22427987 PMC3299753

[68] Lugo-Villarino G Balla KM Stachura DL Bañuelos K Werneck MB Traver D. Identification of dendritic antigen-presenting cells in the zebrafish. Proc Natl Acad Sci U S A (2010) 107(36):15850–5. 10.1073/pnas.1000494107 20733076 PMC2936643

[69] Wittamer V Bertrand JY Gutschow PW Traver D. Characterization of the mononuclear phagocyte system in zebrafish. Blood (2011) 117(26):7126–35. 10.1182/blood-2010-11-321448 21406720

[70] Iger Y Abraham M Dotan A Fattal B Rahamim E Cellular-responses in the skin of carp maintained in organically fertilized water. J Fish Biol (1988) 33(5):711–20. 10.1111/j.1095-8649.1988.tb05516.x

[71] Peleteiro MC Richards RH Phagocytic cells in the epidermis of rainbow-trout, *Salmo gairdneri* Richardson. J Fish Dis (1990) 13(3):225–32. 10.1111/j.1365-2761.1990.tb00777.x

[72] Franch R Cardazzo B Antonello J Castagnaro M Patarnello T Bargelloni L. Full-length sequence and expression analysis of Toll-like receptor 9 in the gilthead seabream (*Sparus aurata* L.). Gene (2006) 378:42–51. 10.1016/j.gene.2006.04.025 16797882

[73] Pietretti D Spaink HP Falco A Forlenza M Wiegertjes GF. Accessory molecules for toll-like receptors in teleost fish. Identification of TLR4 interactor with leucine-rich repeats (TRIL). Mol Immunol (2013) 56(4):745–56. 10.1016/j.molimm.2013.07.012 23958499

[74] Li YW Luo XC Dan XM Huang XZ Qiao W Zhong ZP Orange-spotted grouper (*Epinephelus coioides*) TLR2, MyD88 and IL-1 beta involved in anti-*Cryptocaryon irritans* response. Fish Shellfish Immunol (2011) 30(6):1230–40. 10.1016/j.fsi.2011.04.012 21540114

[75] Zhao LL Liu M Ge JW Qiao XY Li YJ Liu DQ. Expression of infectious pancreatic necrosis virus (IPNV) VP2-VP3 fusion protein in *Lactobacillus casei* and immunogenicity in rainbow trouts. Vaccine (2012) 30(10):1823–9. 10.1016/j.vaccine.2011.12.132 22234263

[76] Sigh J Lindenstrom T Buchmann K. The parasitic ciliate *Ichthyophthirius multifiliis* induces expression of immune relevant genes in rainbow trout, *Oncorhynchus mykiss* (Walbaum). J Fish Dis (2004) 27(7):409–17. 10.1111/j.1365-2761.2004.00558.x 15228610

[77] Peleteiro MC Richards RH Immunocytochemical studies on immunoglobulin-containing cells in the epidermis of rainbow-trout *Salmo-gairdneri* Richardson – influence of bath vaccination. J Fish Biol (1988) 32(6):845–58. 10.1111/j.1095-8649.1988.tb05428.x

[78] Zaccone G Kapoor BG Fasulo S Ainis L Structural, histochemical and functional aspects of the epidermis of fishes. Adv Mar Biol (2001) 40:253–348. 10.1016/S0065-2881(01)40004-6

[79] Asbakk K Elimination of foreign material by epidermal malpighian cells during wound healing in fish skin. J Fish Biol (2001) 58(4):953–66. 10.1111/j.1095-8649.2001.tb00547.x

[80] Sigh J Lindenstrom T Buchmann K. Expression of pro-inflammatory cytokines in rainbow trout (*Oncorhynchus mykiss*) during an infection with *Ichthyophthirius multifiliis*. Fish Shellfish Immunol (2004) 17(1):75–86. 10.1016/j.fsi.2003.12.005 15145419

[81] Lindenstrom T Secombes CJ Buchmann K. Expression of immune response genes in rainbow trout skin induced by *Gyrodactylus derjavini* infections. Vet Immunol Immunopathol (2004) 97(3–4):137–48. 10.1016/j.vetimm.2003.08.016 14741133

[82] Musharrafieh R Tacchi L Trujeque J LaPatra S Salinas I. *Staphylococcus warneri*, a resident skin commensal of rainbow trout (*Oncorhynchus mykiss*) with pathobiont characteristics. Vet Microbiol (2014) 169(1–2):80–8. 10.1016/j.vetmic.2013.12.012 24438987 PMC3967797

[83] Yeh HY Klesius PH. Sequence analysis, characterization and mRNA distribution of channel catfish (*Ictalurus punctatus* Rafinesque, 1818) chemokine (C-X-C motif) receptor 4 (CXCR4) cDNA. Vet Immunol Immunopathol (2010) 134(3–4):289–95. 10.1016/j.vetimm.2009.09.022 19853928

[84] Montero J Garcia J Ordas MC Casanova I Gonzalez A Villena A Specific regulation of the chemokine response to viral hemorrhagic septicemia virus at the entry site. J Virol (2011) 85(9):4046–56. 10.1128/JVI.02519-10 21325404 PMC3126238

[85] Wang XT Clark TG Noe J Dickerson HW. Immunisation of channel catfish, *Ictalurus punctatus*, with *Ichthyophthirius multifiliis* immobilisation antigens elicits serotype-specific protection. Fish Shellfish Immunol (2002) 13(5):337–50. 10.1006/fsim.2001.0410 12458741

[86] Wang XT Dickerson HW. Surface immobilization antigen of the parasitic ciliate *Ichthyophthirius multifiliis* elicits protective immunity in channel catfish (*Ictalurus punctatus*). Clin Diagn Lab Immunol (2002) 9(1):176–81. 10.1128/CDLI.9.1.176-181.2002 11777850 PMC119907

[87] Dickerson H Clark T. *Ichthyophthirius multifiliis*: a model of cutaneous infection and immunity in fishes. Immunol Rev (1998) 166:377–84. 10.1111/j.1600-065X.1998.tb01277.x 9914927

[88] Peleteiro MC Richards RH Identification of lymphocytes in the epidermis of the rainbow trout, *Salmo gairdneri* Richardson. J Fish Dis (1985) 8(2):161–72. 10.1111/j.1365-2761.1985.tb01211.x

[89] Zhang H Shen B Wu H Gao L Liu Q Wang Q Th17-like immune response in fish mucosal tissues after administration of live attenuated *Vibrio anguillarum* via different vaccination routes. Fish Shellfish Immunol (2014) 37(2):229–38. 10.1016/j.fsi.2014.02.007 24561130

[90] Takizawa F Koppang EO Ohtani M Nakanishi T Hashimoto K Fischer U Constitutive high expression of interleukin-4/13A and GATA-3 in gill and skin of salmonid fishes suggests that these tissues form Th2-skewed immune environments. Mol Immunol (2011) 48(12–13):1360–8. 10.1016/j.molimm.2011.02.014 21489635

[91] Overgard AC Nepstad I Nerland AH Patel S. Characterisation and expression analysis of the Atlantic halibut (*Hippoglossus hippoglossus* L.) cytokines: IL-1 beta, IL-6, IL-11, IL-12 beta and IFN gamma. Mol Biol Rep (2012) 39(3):2201–13. 10.1007/s11033-011-0969-x 21643951 PMC3271213

[92] Zhao XG Findly RC Dickerson HW. Cutaneous antibody-secreting cells and B cells in a teleost fish. Dev Comp Immunol (2008) 32(5):500–8. 10.1016/j.dci.2007.08.009 18045689

[93] Boutier M Ronsmans M Ouyang P Fournier G Reschner A Rakus K Rational development of an attenuated recombinant Cyprinid herpesvirus 3 vaccinevsing vrokaryotic vutagenesis and *in vivo* bioluminescent imaging. PLoS Pathog (2015) 11(2):e1004690 10.1371/journal.ppat.1004690 25700279 PMC4336323

[94] Liu X Wu H Chang X Tang Y Liu Q Zhang Y. Notable mucosal immune responses induced in the intestine of zebrafish (*Danio rerio*) bath-vaccinated with a live attenuated *Vibrio anguillarum* vaccine. Fish Shellfish Immunol (2014) 40(1):99–108. 10.1016/j.fsi.2014.06.030 24997435

[95] Dickerson HW Findly RC. Immunity to *Ichthyophthirius* infections in fish: a synopsis. Dev Comp Immunol (2014) 43(2):290–9. 10.1016/j.dci.2013.06.004 23810781

[96] Shibasaki Y Toda F Kobayashi I Moritomo T Nakanishi T. Kinetics of CD4(+) and CD8 alpha(+) T-cell subsets in graft-versus-host reaction (GVHR) in ginbuna crucian carp *Carassius auratus* langsdorfii. Dev Comp Immunol (2010) 34(10):1075–81. 10.1016/j.dci.2010.05.009 20493902

[97] Carballido JM Schols D Namikawa R Zurawski S Zurawski G Roncarolo MG IL-4 induces human B-cell maturation and IgE synthesis in scid-hu mice – inhibition of ongoing IgE production by *in-vivo* treatment with an IL-4/IL-13 receptor antagonist. J Immunol (1995) 155(9):4162–70. 7594571

[98] Defrance T Carayon P Billian G Guillemot JC Minty A Caput D Interleukin-13 is a B-cell stimulating factor. J Exp Med (1994) 179(1):135–43. 10.1084/jem.179.1.135 7903680 PMC2191343

[99] Guo BC Rothstein TL. IL-4 upregulates Ig alpha and Ig beta protein, resulting in augmented IgM maturation and B cell receptor-triggered B cell activation. J Immunol (2013) 191(2):670–7. 10.4049/jimmunol.1203211 23776171 PMC4456092

[100] Lovy J Wright GM Speare DJ Morphological presentation of a dendritic-like cell within the gills of chinook salmon infected with *Loma salmonae*. Dev Comp Immunol (2006) 30(3):259–63. 10.1016/j.dci.2005.06.003 16139356

[101] Doñate C Roher N Balasch JC Ribas L Goetz FW Planas JV CD83 expression in sea bream macrophages is a marker for the LPS-induced inflammatory response. Fish Shellfish Immunol (2007) 23(4):877–85. 10.1016/j.fsi.2007.03.016 17521923

[102] Pettersen EF Ingerslev HC Stavang V Egenberg M Wergeland HI. A highly phagocytic cell line TO from Atlantic salmon is CD83 positive and M-CSFR negative, indicating a dendritic-like cell type. Fish Shellfish Immunol (2008) 25(6):809–19. 10.1016/j.fsi.2008.08.014 18817880

[103] Xu C Evensen O Munang’andu HM. De novo assembly and transcriptome analysis of Atlantic salmon macrophage/dendritic-like TO cells following type I IFN treatment and Salmonid alphavirus subtype-3 infection. BMC Genomics (2015) 16:96. 10.1186/s12864-015-1302-1 25765343 PMC4337061

[104] Hu YH Zhang M Sun L. Expression of *Scophthalmus maximus* CD83 correlates with bacterial infection and antigen stimulation. Fish Shellfish Immunol (2010) 29(4):608–14. 10.1016/j.fsi.2010.06.014 20561589

[105] Olsen MM Kania PW Heinecke RD Skjoedt K Rasmussen KJ Buchmann K. Cellular and humoral factors involved in the response of rainbow trout gills to *Ichthyophthirius multifiliis* infections: molecular and immunohistochemical studies. Fish Shellfish Immunol (2011) 30(3):859–69. 10.1016/j.fsi.2011.01.010 21272651

[106] Alvarez-Pellitero P. Fish immunity and parasite infections: from innate immunity to immunoprophylactic prospects. Vet Immunol Immunopathol (2008) 126(3–4):171–98. 10.1016/j.vetimm.2008.07.013 18783835

[107] Ordás MC Castro R Dixon B Sunyer JO Bjork S Bartholomew J Identification of a novel CCR7 gene in rainbow trout with differential expression in the context of mucosal or systemic infection. Dev Comp Immunol (2012) 38(2):302–11. 10.1016/j.dci.2012.07.001 22858409 PMC3739294

[108] von Gersdorff JL Heinecke RD Skjodt K Rasmussen KJ Buchmann K. Experimental evidence for direct in situ binding of IgM and IgT to early trophonts of *Ichthyophthirius multifiliis* (Fouquet) in the gills of rainbow trout, *Oncorhynchus mykiss* (Walbaum). J Fish Dis (2011) 34(10):749–55. 10.1111/j.1365-2761.2011.01291.x 21916900

[109] Heinecke RD Chettri JK Buchmann K. Adaptive and innate immune molecules in developing rainbow trout, *Oncorhynchus mykiss* eggs and larvae: expression of genes and occurrence of effector molecules. Fish Shellfish Immunol (2014) 38(1):25–33. 10.1016/j.fsi.2014.02.010 24561127

[110] Heinecke RD Buchmann K. Inflammatory response of rainbow trout *Oncorhynchus mykiss* (Walbaum, 1792) larvae against *Ichthyophthirius multifiliis*. Fish Shellfish Immunol (2013) 34(2):521–8. 10.1016/j.fsi.2012.11.036 23261502

[111] Lin SH Davidson GA Secombes CJ Ellis AE A morphological study of cells isolated from the perfused gill of dab and Atlantic salmon. J Fish Biol (1998) 53(3):560–8. 10.1111/j.1095-8649.1998.tb01001.x

[112] Mulero I Pilar Sepulcre M Roca FJ Meseguer J García-Ayala A Mulero V. Characterization of macrophages from the bony fish gilthead seabream using an antibody against the macrophage colony-stimulating factor receptor. Dev Comp Immunol (2008) 32(10):1151–9. 10.1016/j.dci.2008.03.005 18420271

[113] Barnett RR Akindele T Orte C Shephard KL Eosinophilic granulocytes in the epidermis of *Oreochromis mossambicus* gill filaments studied in situ. J Fish Biol (1996) 49(1):148–56. 10.1006/jfbi.1996.0142

[114] Mulero I Sepulcre MP Meseguer J Garcia-Ayala A Mulero V. Histamine is stored in mast cells of most evolutionarily advanced fish and regulates the fish inflammatory response. Proc Natl Acad Sci U S A (2007) 104(49):19434–9. 10.1073/pnas.0704535104 18042725 PMC2148307

[115] Buonocore F Randelli E Tranfa P Scapigliati GA. CD83-like molecule in sea bass (*Dicentrarchus labrax*): molecular characterization and modulation by viral and bacterial infection. Fish Shellfish Immunol (2012) 32(6):1179–84. 10.1016/j.fsi.2012.02.027 22554578

[116] Wang K Mu Y Qian T Ao J Chen X. Molecular characterization and expression analysis of toll-like receptor 1 from large yellow croaker (*Pseudosciaena crocea*). Fish Shellfish Immunol (2013) 35(6):2046–50. 10.1016/j.fsi.2013.10.022 24184976

[117] Taechavasonyoo A Kondo H Nozaki R Suzuki Y Hirono I. Identification of novel interleukin 1 beta family genes in Japanese flounder *Paralichthys olivaceus*. Fish Shellfish Immunol (2013) 34(1):393–6. 10.1016/j.fsi.2012.10.001 23069785

[118] Taechavasonyoo A Hirono I Kondo H. The immune-adjuvant effect of Japanese flounder *Paralichthys olivaceus* IL-1beta. Dev Comp Immunol (2013) 41(4):564–8. 10.1016/j.dci.2013.07.003 23850723

[119] Dan XM Zhang TW Li YW Li AX. Immune responses and immune-related gene expression profile in orange-spotted grouper after immunization with *Cryptocaryon irritans* vaccine. Fish Shellfish Immunol (2013) 34(3):885–91. 10.1016/j.fsi.2012.12.011 23291105

[120] Mladineo I Block BA. Expression of cytokines IL-1 beta and TNF-alpha in tissues and cysts surrounding *Didymocystis wedli* (Digenea, Didymozoidae) in the Pacific bluefin tuna (*Thunnus orientalis*). Fish Shellfish Immunol (2010) 29(3):487–93. 10.1016/j.fsi.2010.05.008 20580835

[121] Covello JM Bird S Morrison RN Battaglene SC Secombes CJ Nowak BF. Cloning and expression analysis of three striped trumpeter (*Latris lineata*) pro-inflammatory cytokines, TNF-alpha, IL-1 beta and IL-8, in response to infection by the ectoparasitic, *Chondracanthus goldsmidi*. Fish Shellfish Immunol (2009) 26(5):773–86. 10.1016/j.fsi.2009.03.012 19332136

[122] Abos B Castro R Luque A Gonzalez L Tafalla C Heterogenity of IgM cells in rainbow trout (*Oncorhynchus mykiss*) tissues. Fish Shellfish Immunol (2013) 34(6):1636 10.1016/j.fsi.2013.03.007 23257202

[123] Abós B Castro R Pignatelli J Luque A González L Tafalla C. Transcriptional heterogeneity of IgM(+) Cells in rainbow trout (*Oncorhynchus mykiss*) tissues. PLoS One (2013) 8(12):e82737. 10.1371/journal.pone.0082737 24324826 PMC3855791

[124] Nuñez Ortiz N Gerdol M Stocchi V Marozzi C Randelli E Bernini C T cell transcripts and T cell activities in the gills of the teleost fish sea bass (*Dicentrarchus labrax*). Dev Comp Immunol (2014) 47(2):309–18. 10.1016/j.dci.2014.07.015 25109574

[125] Harun NO Wang T Secombes CJ. Gene expression profiling in naive and vaccinated rainbow trout after *Yersinia ruckeri* infection: insights into the mechanisms of protection seen in vaccinated fish. Vaccine (2011) 29(26):4388–99. 10.1016/j.vaccine.2011.04.003 21504776

[126] Chang YT Kai YH Chi SC Song YL. Cytotoxic CD8 alpha(+) leucocytes have heterogeneous features in antigen recognition and class I MHC restriction in grouper. Fish Shellfish Immunol (2011) 30(6):1283–93. 10.1016/j.fsi.2011.03.018 21463694

[127] Somamoto T Miura Y Nakanishi T Nakao M. Local and systemic adaptive immune responses toward viral infection via gills in ginbuna crucian carp. Dev Comp Immunol (2015) 52(1):81–7. 10.1016/j.dci.2015.04.016 25936589

[128] Aquilino C Castro R Fischer U Tafalla C. Transcriptomic responses in rainbow trout gills upon infection, with viral hemorrhagic septicemia virus (VHSV). Dev Comp Immunol (2014) 44(1):12–20. 10.1016/j.dci.2013.11.006 24269609

[129] LaPatra S Kao S Erhardt EB Salinas I. Evaluation of dual nasal delivery of infectious hematopoietic necrosis virus and enteric red mouth vaccines in rainbow trout (*Oncorhynchus mykiss*). Vaccine (2015) 33(6):771–6. 10.1016/j.vaccine.2014.12.055 25562788

[130] Iliev DB Thim H Lagos L Olsen R Jorgensen JB. Homing of antigen presenting cells in headkidney and spleen – salmon headkidney hosts diverse APC types. Front Immunol (2013) 4:137. 10.3389/fimmu.2013.00137 23761795 PMC3674399

[131] Jorgensen LV Heinecke RD Skjodt K Rasmussen KJ Buchmann K. Experimental evidence for direct in situ binding of IgM and IgT to early trophonts of *Ichthyophthirius multifiliis* (Fouquet) in the gills of rainbow trout, *Oncorhynchus mykiss* (Walbaum). J Fish Dis (2011) 34(10):749–55. 10.1111/j.1365-2761.2011.01291.x 21916900

[132] Howarth M Elliott T. The processing of antigens delivered as DNA vaccines. Immunol Rev (2004) 199(1):27–39. 10.1111/j.0105-2896.2004.00141.x 15233724

[133] Munang’andu HM Mutoloki S Evensen O Non-replicating vaccines. Fish Vaccin (2014) 3:22–32. 10.1002/9781118806913.ch3

[134] Munang’andu HM Sandtrø A Mutoloki S Brudeseth BE Santi N Evensen Ø. Immunogenicity and cross protective ability of the central VP2 amino acids of infectious pancreatic necrosis virus in Atlantic salmon (*Salmo salar* L.). PLoS One (2013) 8(1):e54263. 10.1371/journal.pone.0054263 23349841 PMC3549989

[135] Gadan K Sandtrø A Marjara IS Santi N Munang’andu HM Evensen Ø. Stress induced reversion to virulence of infectious pancreatic necrosis virus in naive fry of Atlantic salmon (*Salmo salar* L.). PLoS One (2013) 8(2):e54656. 10.1371/journal.pone.0054656 23431359 PMC3576400

